# Unveiling the Differential Antioxidant Activity of Maslinic Acid in Murine Melanoma Cells and in Rat Embryonic Healthy Cells Following Treatment with Hydrogen Peroxide

**DOI:** 10.3390/molecules25174020

**Published:** 2020-09-03

**Authors:** Khalida Mokhtari, Amalia Pérez-Jiménez, Leticia García-Salguero, José A. Lupiáñez, Eva E. Rufino-Palomares

**Affiliations:** 1 Department of Biochemistry and Molecular Biology I, Faculty of Sciences, University of Granada, Avenida Fuentenueva, s/n, 18071 Granada, Spain; khalidafadoua@hotmail.com (K.M.); elgarcia@ugr.es (L.G.-S.); jlcara@ugr.es (J.A.L.); 2 Laboratory of Bioresources, Biotechnologies, Ethnopharmacology and Health, Department of Biology, Faculty of Sciences, Mohammed I University of Oujda, Oujda 60000, Morocco; 3 Department of Zoology, Faculty of Sciences, University of Granada, Avenida Fuentenueva, s/n, 18071 Granada, Spain; calaya@ugr.es

**Keywords:** antioxidant activity, antioxidant enzymes, anti-proliferative activity, maslinic acid, melanoma, *Olea europaea* L., ROS levels

## Abstract

Maslinic acid (MA) is a natural triterpene from *Olea europaea* L. with multiple biological properties. The aim of the present study was to examine MA’s effect on cell viability (by the MTT assay), reactive oxygen species (ROS levels, by flow cytometry) and key antioxidant enzyme activities (by spectrophotometry) in murine skin melanoma (B16F10) cells compared to those on healthy cells (A10). MA induced cytotoxic effects in cancer cells (IC_50_ 42 µM), whereas no effect was found in A10 cells treated with MA (up to 210 µM). In order to produce a stress situation in cells, 0.15 mM H_2_O_2_ was added. Under stressful conditions, MA protected both cell lines against oxidative damage, decreasing intracellular ROS, which were higher in B16F10 than in A10 cells. The treatment with H_2_O_2_ and without MA produced different responses in antioxidant enzyme activities depending on the cell line. In A10 cells, all the enzymes were up-regulated, but in B16F10 cells, only superoxide dismutase, glutathione S-transferase and glutathione peroxidase increased their activities. MA restored the enzyme activities to levels similar to those in the control group in both cell lines, highlighting that in A10 cells, the highest MA doses induced values lower than control. Overall, these findings demonstrate the great antioxidant capacity of MA.

## 1. Introduction

An imbalance between pro-oxidant and antioxidant molecules can lead to an oxidative stress situation that modifies normal cell physiology due to protein, lipid, carbohydrate and nucleic acid damage [1]. Many cellular processes depend on the variations in the levels of ROS and NADPH that take place during their development and that, fundamentally, are determined by the activity of the different production systems for this reduced coenzyme, especially those belonging to the pentose phosphate pathway (PPP), glucose-6-phosphate dehydrogenase (G6PDH), 6-phosphogluconate dehydrogenase (6PGDH) and, also, NADP-dependent isocitrate dehydrogenase (ICDH-NADP). The cellular redox state is key to interpreting the behavior of most of these key cellular processes for the vital development of organisms, such as cell differentiation [2,3,4], cellular growth [5,6,7,8], cell nutrition [6,9,10,11] and cell aging [12].

Reactive oxygen species (ROS) and reactive nitrogen species (RNS) are the most important endogenous pro-oxidants produced by normal cellular metabolism [13]. Under a normal physiological situation, an antioxidant defense system neutralizes ROS. This antioxidant system involves enzymes such as catalase (CAT), which reduces hydrogen peroxide; superoxide dismutase (SOD), which detoxifies the superoxide radical; glutathione peroxidase (GPX), which reduces hydrogen peroxide and other organic peroxides; S-transferase glutathione (GST), which detoxifies harmful molecules; glutathione reductase (GR), which regenerates glutathione (GSH) from its oxidized form (GSSG) by an NADPH-dependent pathway; and G6PDH, which produces NADPH for GR’s mechanism. Beside enzymes, other molecules can also act as antioxidants, such as NADPH, GSH and different vitamins, among other molecules [14,15,16]. Despite this antioxidant system, an excessive production of ROS can lead to an oxidative stress situation inducing several types of damage to different biomolecules [17]. In cancer processes, an excessive production of ROS has been related to genomic instability due to the induction of DNA damage and to the alterations in signaling pathways involved in survival, proliferation and apoptosis resistance. Moreover, it has been demonstrated that ROS induce vascular endothelial growth factor (VEGF) expression, producing neovascularization and fast expansion of the cancer, modifying angiogenesis and metastasis, which induce the malignancy of cancer cells [17]. The use of natural compounds with antioxidant capacity that reduce ROS levels could decrease metastatic progression [15,17]. Furthermore, the immune defense system can be altered by ROS, since high levels of these molecules produced by NADPH oxidase can inhibit monocytes and macrophages and weaken the response of T-cells due to the down-regulation of several cytokines [17].

Hydrogen peroxide (H_2_O_2_) is originated from O_2_ by SOD. It is not a free radical as such, but it is a reactive molecule, since it has the capacity to generate the hydroxyl radical in the presence of metals such as iron [1]. H_2_O_2_ is an important metabolite that arises mainly during aerobic metabolism, although it can derive from other sources [18]. H_2_O_2_ is a messenger molecule that diffuses through cells and tissues, inducing different effects that include deformations of the shapes of cells, the initiation of proliferation and the recruitment of immune cells. If not controlled, an excess of H_2_O_2_ can cause uncontrolled oxidative stress in cells, producing irreparable damage [19]. Therefore, cell death and survival processes can be modulated by controlling intracellular H_2_O_2_ levels by the use of antioxidants. Natural products are gaining interest due to their cost-effectiveness, few side effects and high availability. As such, products such as maslinic acid (MA) have received heightened interest. 

**Figure 1:**
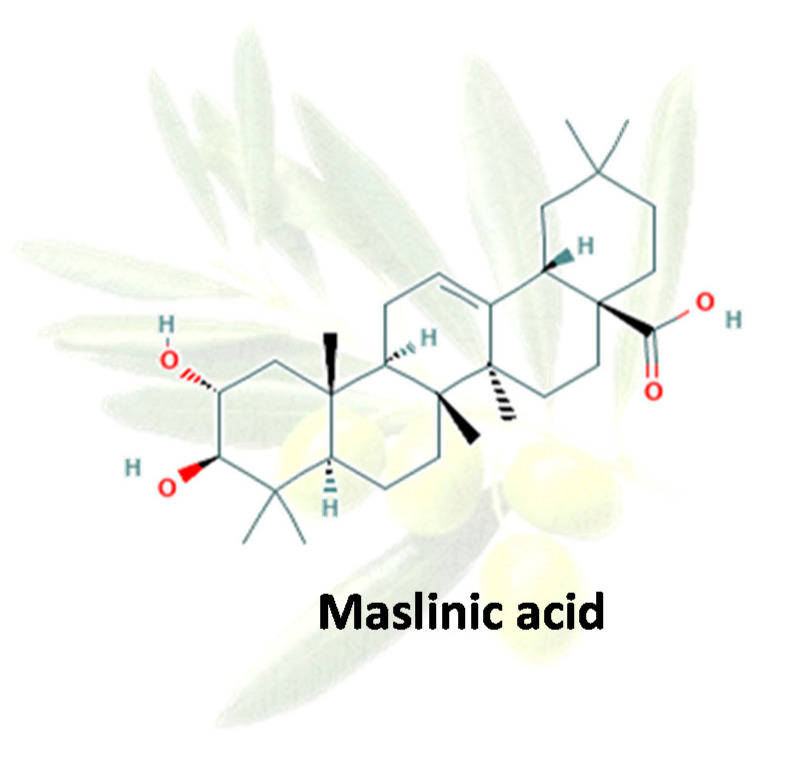
Schedule chemical structure of maslinic acid from pomace olive (*Olea europaea* L.) (PubChem).

MA, C_30_H_48_O_4_ (2α,3β-dihydroxyolean-12-en-28-oic acid) is a natural pentacyclic triterpene, also known as crataegolic acid and derived from its structural analogue, oleanolic acid (Figure 1). It is formed by 30 carbon atoms grouped into five cycles that, as substituents, have two methyl groups each on the C-4 and C-21 carbons; single methyl groups on the C-8, C-10 and C-15 carbons; two hydroxyl groups each on the C-2 and C-3 carbons; one carboxyl group on the C-28 carbon; and a double bond on the C-12 and C-13 [20,21,22,23].

Maslinic acid is present in various plants, many of them common in the Mediterranean diet, such as eggplants, spinach, lentils, chickpeas, and even different aromatic herbs [24]. Moreover, it is especially abundant in *Crataegus oxyacantha*, in the surface wax of the fruits and leaves of *Olea europaea* L. and in the solid waste from olive oil extraction [25]. Furthermore, MA is among the main triterpenes present in olives and olive oil. Its concentration in the oil depends on the type of olive oil and the variety of olive tree [23].

MA enjoys high pharmacological interest, owing to its anti-tumor effect in certain types of cancer besides its anti-inflammatory, antioxidant, anti-diabetogenic, anti-hypertensive, anti-viral and cardioprotective effects, among others [1,23]. Among the bioactivities attributed to MA, its antioxidant effect is the most contradictory. It has been observed that the oxidative status induced by CCl_4_ was decreased by MA treatment, which reduced lipid peroxides in the plasma and the susceptibility of lipids to peroxidation [26]. In the same way, the LDL oxidation in the plasma of rabbits produced by CuSO^4-^ was decreased by MA obtained from *Punica granatum* [27]. In human plasma, MA showed peroxyl-radical scavenging and chelating capacities for copper but did not show a positive effect for the prevention of LDL oxidation [28]. Research in macrophages revealed that MA can act in a similar way to catalase, decreasing the generation of H_2_O_2_, but does not produce any direct inhibitory effects on NO and superoxide formation [29]. In a previous study, we concluded that MA produced an increase in the ROS level under stress conditions caused by the absence of FBS in melanoma cells [15]. Furthermore, in this same study, MA had an antioxidant effect at lower assayed levels; however, at higher dosages, MA induced cellular damage by apoptosis.

The aim of the present study was to evaluate MA’s antioxidant effects in murine skin melanoma (B16F10) cells and in a healthy cell line derived from the thoracic aorta of an embryonic rat (A10), by analyzing the proliferation, ROS production and the activity of the main antioxidant enzymes under cellular stress conditions induced by H_2_O_2_.

## 2. Results

### 2.1. MA Decreases Proliferation of B16F10 Cells by a Dose-Dependent Mechanism

**Figure 2:**
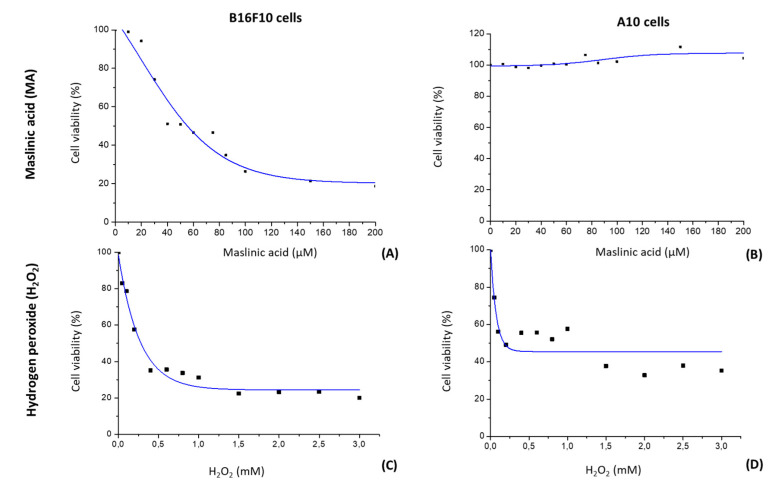
Cytotoxicity curves of maslinic acid (MA) for B16F10 murine melanoma cells (**A**) and A10 rat embryonic healthy cells (**B**) and of hydrogen peroxide (H_2_O_2_) for B16F10 murine melanoma cells (**C**) and A10 rat embryonic healthy cells (**D**). Cell proliferation was determined by the MTT assay. Values are expressed as means ± SEM (*n* = 9).

We evaluated MA’s effect on B16F10 melanoma and A10 cell line proliferation using the MTT assay (Figure 2A,B). B16F10 and A10 cells were exposed to different doses of MA (0 to 212 μM) for 24 h. Then, cell survival was compared with that of untreated control cells. The percentage of living cells decreased as the dose of MA increased in cancer cells (B16F10), while in healthy cells (A10), MA did not produce any cytotoxic effect, even at the highest doses used (210 µM). In B16F10 cells, the growth inhibition values (IC_50_) in response to MA were 42 µM after 24 h of addition of this compound. Based on this value, the rest of studies were performed with an exposure of cells to 10.6 μM (IC_50/4_), 21.2 μM (IC_50/2_), 42.3 μM (IC_50_) and 84.6 μM (2·IC_50_) of MA for 24 h.

### 2.2. H_2_O_2_ Modifies Cell Viability

We examined H_2_O_2_’s effects (0 to 3 mM of H_2_O_2_ for 24 h) on the cell viability of B16F10 and A10 cells to determine the optimal dose capable of producing stress without inducing cell death (Figure 2C,D). Cell survival was compared with that of untreated controls. The percentage of living cells decreased as the dose of H_2_O_2_ increased in both cell lines up to the concentration of 1.5 mM, beyoond which H_2_O_2_ had no cell viability effect. In both cell lines, we noticed an IC_50_ of 0.2 mM. In no case, with the doses used and the incubation time employed, was any mortality higher than 80% reached. Based on these results, the concentration of 0.15 mM of H_2_O_2_ was used to perform the rest of the studies.

### 2.3. Maslinic Acid’s Influence on Mitochondrial-Membrane Potential

**Figure 3:**
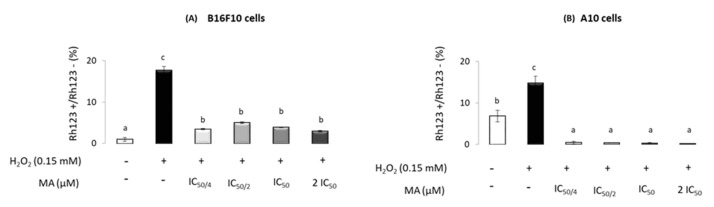
Positive fluorescent Rh123 on B16F10 cells (**A**) and A10 cells (**B**) with (+) or without (−) H_2_O_2_ and MA treatment at different doses: IC_50/4_, IC_50/2_, IC_50_ and 2·IC_50_ (10.6, 21.6, 42.3 and 84.6 μM, respectively). Values are expressed as means ± SEM (*n* = 9). Different letters indicate significant differences (*p* < 0.05).

An assay of ROS production was performed to test the ROS levels that occurred over time in the presence of 0.150 mM H_2_O_2_ and different MA levels (IC_50/4_, IC_50/2_, IC_50_ and 2·IC_50_). The results are shown in Figure 3. After 24 h of H_2_O_2_ treatment, ROS levels increased significantly in both cell lines, compared to those in the controls. The increment observed upon H_2_O_2_ addition was offset by MA supplementation, which resulted in decreased intracellular ROS levels at any MA level used (IC_50/4_, IC_50/2_, IC_50_ and 2·IC_50_) in B16F10 and A10 cells. Moreover, in A10, ROS levels decreased below the value of control cells for all concentrations of MA tested.

### 2.4. MA Exerts Antioxidant Activity, Modulating Enzymatic Defense System

**Figure 4:**
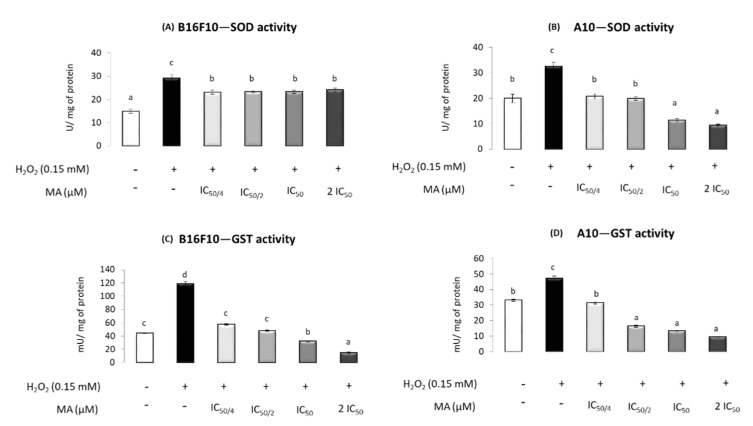
Effect of different maslinic acid doses—IC_50/4_, IC_50/2_, IC_50_ and 2·IC_50_ (10.6, 21.6, 42.3 and 84.6 μM, respectively)—on the specific activity of superoxide dismutase (SOD) in cancer cells, B16F10 (**A**), and normal cells, A10 (**B**), and glutathione S-transferase (GST) in cancer cells, B16F10 (**C**), and normal cells, A10 (**D**), subjected to the presence of hydrogen peroxide. Symbols (+) and (−) indicate the presence or absence of incubation with H_2_O_2_, respectively. Enzyme activities (U or mU × mg protein^−1^) are expressed as means ± SEM (*n* = 9). Different letters indicate significant differences (*p* < 0.05).

The results obtained for SOD in B16F10 cells showed that H_2_O_2_ increased its activity and MA (at any level) decreased it, but without significant differences induced by different MA concentrations. In A10, H_2_O_2_ increased the SOD activity, an increment that was mitigated with MA addition. Moreover, 42.3 and 84.6 μM MA (IC_50_ and 2·IC_50_, respectively) resulted in SOD activity levels lower than those found in the absence of H_2_O_2_ and MA (Figure 4A,B).

In B16F10 cells, GST activity increased in the presence of H_2_O_2_, whereas the incubation with the IC_50/4_ and IC_50/2_ of MA decreased the levels, making them equal to those in the control without H_2_O_2_ and MA. The use of the IC_50_ and 2·IC_50_ MA concentrations induced GST activities lower than those in the control. Similar results were found in A10 for GST activity, with the exception of MA at IC_50/2_, which also induced lower levels than control (Figure 4C,D).

**Figure 5:**
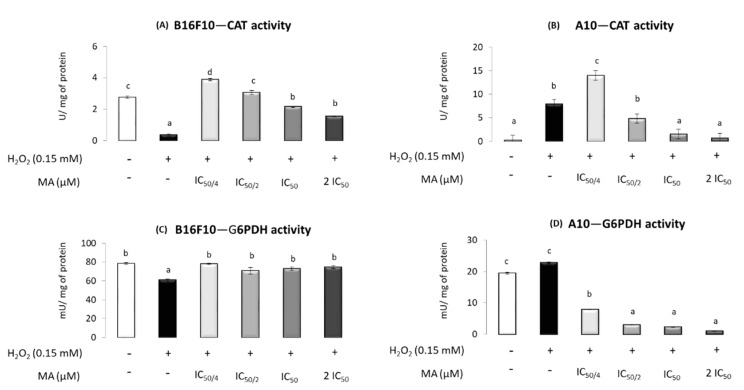
Effect of different maslinic acid doses—IC_50/4_, IC_50/2_, IC_50_ and 2·IC_50_ (10.6, 21.6, 42.3 and 84.6 μM, respectively)—on the specific activity of catalase (CAT) in cancer cells, B16F10 (**A**), and normal cells, A10 (**B**), and glucose 6-phosphate dehydrogenase (G6PDH) in cancer cells, B16F10 (**C**), and normal cells, A10 (**D**), subjected to the presence of hydrogen peroxide. Symbols (+) and (−) indicate the presence or absence of incubation with H_2_O_2_, respectively. Enzyme activities (U or mU × mg protein^−1^) are expressed as means ± SEM (*n* = 9). Different letters indicate significant differences (*p* < 0.05).

In B16F10 cells, CAT activity decreased in the presence of H_2_O_2_ compared to that in the control, whereas MA increased the activity, inducing values higher than or similar to control when IC_50/4_ or IC_50/2_ were used. In A10 cells, H_2_O_2_ produced an increase in CAT activity, and its activity was not recovered until the highest doses of MA were used (IC_50_ and 2·IC_50_) (Figure 5A,B).

Regarding G6PDH, the results indicated its activity decreased in the presence of H_2_O_2_ in B16F10 and MA at all the concentrations tested, with similar values to the control without H_2_O_2_ and MA. In A10 cells, no changes were induced by H_2_O_2_ in G6PDH, whereas MA decreased its activity at any tested level (Figure 5C,D).

**Figure 6:**
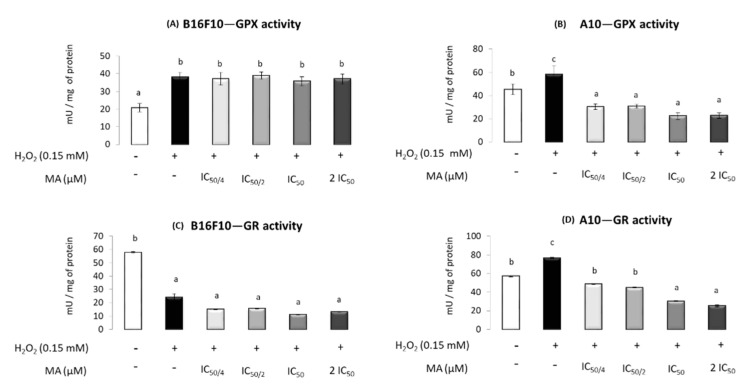
Effect of different maslinic acid doses—IC_50/4_, IC_50/2_, IC_50_ and 2·IC_50_ (10.6, 21.6, 42.3 and 84.6 μM, respectively)—on the specific activity of glutathione peroxidase (GPX) in cancer cells, B16F10 (**A**), and normal cells, A10 (**B**), and glutathione reductase (GR) in cancer cells, B16F10 (**C**), and normal cells, A10 (**D**), subjected to the presence of hydrogen peroxide. Symbols (+) and (−) indicate the presence or absence of incubation with H_2_O_2_, respectively. Enzyme activity (mU × mg protein^−1^) is expressed as means ± SEM (*n* = 9). Different letters indicate significant differences (*p* < 0.05)

GPX increased in the presence of H_2_O_2_ but no change was observed when MA was administrated in B16F10. In A10 cells, all concentrations of MA decreased the GPX activity increased by the H_2_O_2_, even below that in the control without H_2_O_2_ and MA (Figure 6A,B).

In B16F10, GR activity was decreased by H_2_O_2_ and no changes were observed at any MA level, with lower levels of activity maintained compared to the control. In A10, H_2_O_2_ increased GR activity, an increment that was mitigated with MA addition. Moreover, 42.3 and 84.6 μM MA induced values of GR activity lower than those found in the absence of H_2_O_2_ and MA (Figure 6C,D).

## 3. Discussion

Maslinic acid (MA) is a pentacyclic triterpene abundant in the surface wax of fruits and leaves of *Olea europaea* L. with many demonstrated biological activities [23,30,31]. For this reason, MA is appreciated as a chemopreventive agent in different diseases such as cancer [23,28], cardiovascular and neurodegenerative pathologies, etc. [26].

In different cell lines, MA’s cytotoxic effect has been studied, including in both cancer and healthy cells. In this sense, it has been shown that this triterpene affects cells in different ways depending on the kind of cells and the conditions of the experiment. In the present study, MA showed cytotoxic effects only in B16F10 cells, and this effect was important, as the IC_50_ of MA was 42 µM. In another study performed in melanoma cells by Kim et al. [32], the authors observed a lower MA cytotoxicity effect. Thus, they concluded that the IC_50_ for MA in SK-MEL-3 was also 42 µM, but in their study, the incubation period was 48 h versus the 24 h used in the present study. Other results obtained in our research group showed that in colon cancer cells, the IC_50_ value for MA was 30 µM in HT29 cells after 72 h of incubation [31,33,34], showing a higher cytotoxic effect in Caco-2 cells, in which the IC_50_ of MA was 10.82 µM, also after 72 h [35]. Other authors observed IC_50_ values for MA ranging between 32 and 64 µM in different cancer cells such as lung (A549), ovary (SK-OV-3), colon (HCT15) and glioma (XF498) cells after 48 h of incubation [32]. In several bladder cancer cell lines incubated with MA for 48 h, other authors found IC_50_ values between 20 and 300 µM [36]. Notwithstanding this, compared to in melanoma cells, a non-cytotoxic effect was found in A10 healthy cells in the present study. These results are relevant since they demonstrate the selective cytotoxic effect of this compound. Studies focused on the selective effect of MA are scarce. Reyes et al. [22] observed that epithelial intestinal cells incubated with 30 µM MA for 72 h (IC_50_ value for HT29) exhibited a survival rate of 78% for IEC-6 cells and 68% for IEC-18.

An intracellular redox balance is crucial to ensure viability, growth and the diversity of cell functions [15]. An excess of ROS is related to pathological processes producing oxidative damage when the antioxidant defense system is not able to counteract it. Hence, there is a growing interest of the scientific community and industry in obtaining substances of natural origin aimed at preventing these pathologies and alterations caused by ROS. In this context, it is important to characterize the protective biochemical functions of natural antioxidants and to study their intracellular pathway signaling. A large number of plant constituents, such as MA, have antioxidant properties [37].

The present study examined the antioxidant effects of MA in skin melanoma cancer cells (B16F10) and thoracic aorta of embryonic rat cells (A10). The major findings were that MA improves the oxidative stress caused by a H_2_O_2_ excess, decreasing the intracellular ROS levels in both cell lines. H_2_O_2_ was used in this study because it is known that when present in excess, it is one of the major compounds that can damage cells [38]. Other authors have clearly shown the effects of H_2_O_2_ on the viability and ROS production of different cell types that were subsequently treated with other antioxidant natural compounds that reversed the oxidative damage caused by the H_2_O_2_ [39,40]. Furthermore, studies similar to ours with other ROS-producing molecules (i.e., CCl_4_) observed that MA counteracted the lipid peroxidation generated in the nucleus [26]. Similarly, MA prevented the CuSO_4_^−^-induced oxidation of rabbit plasma LDL [41]. Moreover, Yang et al. [42] evaluated the antioxidant effects of MA derivatives that showed radical-scavenging activity and inhibition of NO production in RAW 264.7 cells.

Regarding MA’s effect on antioxidant enzymes in the B16F10 cell line, as expected, SOD, GST and GPX activities were increased in response to the H_2_O_2_ addition. However, H_2_O_2_ decreased CAT, G6PDH and GR activities. A H_2_O_2_ excess is responsible for cellular damage that includes an imbalance in membranes and biomolecule alterations. This fact supposes an extra energetic cost for the maintenance of cellular homeostasis in order to repair the damage produced. Both cellular damage and energy requirements could affect G6PDH activity, by either the impaired glucose transporters or enhanced glycolysis pathway, decreasing the glucose available for the pentose phosphate pathway. The regulation of NADPH levels is essential for understanding the behavior of numerous physiological processes, being especially important for growth and cell differentiation [2] but also for antioxidant processes, among others. NAPDH is mainly generated by the G6PDH enzyme and is required for the GPX and CAT activity involved in the H_2_O_2_ removal pathway. Thus, CAT is protected from inactivation by NADPH. Moreover, this reduction equivalent is used by GR to regenerate the oxidized glutathione to its reduced form required for GPX activity [43,44]. The lower G6PDH activity observed when H_2_O_2_ was added could result in a decrease in available NADPH levels, which might justify the reduction in CAT and GR activities. Similar results have been observed in previous studies in B16F10 cells subjected to stress conditions induced by FBS absence [15]. Mokhtari et al. [15] reported low activity levels for CAT that were produced by low NADPH levels due to an imbalance in cellular homeostasis.

It has been established that MA is a compound with antioxidant capacity [15,26,28]. When MA was added to the B16F10 cells, the changes induced by H_2_O_2_ excess in SOD and GST activities were counteracted. In this sense, the scavenger MA’s effects reduced ROS levels and, subsequently, the need for the action of these antioxidant defenses. Moreover, the observed decrease in ROS levels due to MA would result in less cellular damage, which would reverse the possible mechanisms responsible for the decrease in G6PDH when H_2_O_2_ is added, raising its activity up to the control values. This recovered G6PDH activity would produce the NADPH level required to prevent and reverse the down-regulation of CAT [43]. Finally, a dose-response effect was observed for both GPX and CAT, highlighting the antioxidant behavior of MA in these cells.

The results found in this work, in A10 healthy cells, revealed that all the enzyme activities increased in the stressful conditions originated by the H_2_O_2_ addition, except for G6PDH, whose activity was slightly higher. MA, in these cells, restored the antioxidant enzyme activities, inducing values similar to those in the control group in cells treated with the lowest MA levels (IC_50/4_). Furthermore, when cells were treated with the two highest doses of MA (IC_50_ and 2·IC_50_), the activity of all antioxidant enzymes, with the exception of CAT, decreased below control levels. This fact confirms the relevant antioxidant effect of MA [15,28,43].

## 4. Materials and Methods 

### 4.1. Compounds

Maslinic acid was obtained from olive pomace and kindly donated by Biomaslinic S.A., Granada, Spain. The extract is a chemically pure white powder composed of 98% maslinic acid and stable when stored at 4 °C. It was dissolved before use at 10 mg/mL in 50% dimethyl sulfoxide (DMSO) and 50% phosphate buffer solution (PBS). This solution was diluted in cell culture medium for assay purposes.

### 4.2. Cell Lines and Cultures

The mouse melanoma cell line B16F10 is a variant of the murine melanoma cell line B16. These cells show a higher metastatic potential than B16 cells [45]. A10 is a cell line derived from the thoracic aorta of an embryonic mouse, and it is used as a study model of smooth muscle cells (SMC). This cell type shows a great proliferative capacity and may be subcultured several times, allowing the rapid attainment of cell mass [46]. Both cell lines used were provided by the cell bank of the University of Granada (Spain). The cell lines were cultured in Dulbecco’s modified Eagle’s medium (DMEM) containing glucose (4.5 g/L) and L-glutamine (2 mM) from the commercial PAA brand, 10% heat-inactivated fetal bovine serum (FBS), and 0.5% gentamicin for B16F10 cells and 10,000 units/mL penicillin and 10 mg/mL streptomycin for A10 cells. The cell lines were maintained in a humidified atmosphere with 5% CO_2_ at 37 °C. The cells were passaged at preconfluent densities by the use of a solution containing 0.05% trypsin and 0.5 mM EDTA. The cells were seeded in the culture dishes at the desired density. After 24 h, when the cells were attached to the dish, the cells were incubated with 0.15 mM hydrogen peroxide (H_2_O_2_), in order to produce a stress situation in the cells. Following that, the cells were incubated with several concentrations of MA as indicated below.

### 4.3. MTT Assay

The MTT assay was performed as described by Mokhtari et al. [15]. Briefly, a 200 µL cell suspension of B16F10 or A10 (1.5 × 10^3^ cells/well) was cultured in 96-well plates. Subsequent to the adherence of the cells, different MA dilutions on a scale of 10 to 210 µM were added separately. The incubation times were 24 h for all cases. MTT was dissolved in the medium and added to the wells at a final concentration of 0.5 mg/mL. Following 2 h of incubation, the generated formazan was dissolved in DMSO. Absorbance was measured at 570 nm in a multiplate reader (Bio-tek^®^). The absorbance was proportional to the number of viable cells. The MA concentration leading to 50% inhibition of cell proliferation (IC_50_) was determined. The results are expressed as the percentage of live cells compared with the control considered as 100% cell viability. Cell viability in B16F10 and A10 cells was also studied in the presence of H_2_O_2_ by the MTT assay. H_2_O_2_ was dissolved in the culture medium of the cell lines. The concentrations used were made extemporaneously and protected from light before use to prevent degradation of the compounds. After 24 h of incubation, the medium was removed; fresh medium was added with different concentrations of H_2_O_2_ per well—0, 0.05, 0.1, 0.2, 0.4, 0.6, 0.8, 1.1, 1.5, 2.0, 2.6 and 3 mM—to a final volume of 200 µL for 24 h.

### 4.4. Flow-Cytometry Analysis of the Mitochondrial-Membrane Potential

Changes in the mitochondrial-membrane potential can be examined by monitoring the cell fluorescence after double staining with rhodamine 123 (Rh123) and propidium iodide (PI). Rh123 is a membrane-permeable fluorescent cationic dye that is selectively taken up by mitochondria in direct proportion to the MMP (mitochondrial-membrane permeabilization) [47]. B16F10 and A10 cells (4 × 10^5^ cells/well) were seeded on 12-well plates with 2 mL of medium and treated with 0.15 mM H_2_O_2_ for 24 h and MA at IC_50/4_, IC_50/2_, IC_50_ and 2·IC_50_ (10.6, 21.6, 42.3 and 84.6 μM, respectively) concentrations for 24 h more. Following the treatment, the medium was removed and fresh medium with dihydrorhodamine (DHR), at a final concentration of 5 μg/mL, was added. After 30 min of incubation, the medium was removed and the cells were washed and resuspended in PBS with 5 μg/mL of PI. The intensity of fluorescence from Rh123 and PI was determined using an ACS flow cytometer (Coulter Corporation, Hialeah, FL, USA), at the excitation and emission wavelengths of 500 and 536 nm, respectively. The experiments were performed three times with two replicates per assay.

### 4.5. Antioxidant Enzyme Assays

In order to prepare the samples for analytic procedures, cells were homogenized in RIPA buffer. Immediately, the cells were sonicated on ice for 5 min and maintained under moderate shaking at 4 °C for 1 h. Every 15 min, the samples were moderately shaken in a vortex. The lysates were spun in a centrifuge at 10,000× *g* at 4 °C for 15 min. All the enzyme assays were carried out at 37 °C using a Power Wave X microplate scanning spectrophotometer (Bio-Tek Instruments, Winooski, VT, USA) and run in duplicate in 96-well microplates. The optimal substrate and protein concentrations for the measurement of maximal activity for each enzyme were established by preliminary assays. The enzymatic reactions were initiated by the addition of the cell extract, except for SOD, where xanthine oxidase was used. The millimolar extinction coefficients used for H_2_O_2_, NADH/NADPH and DTNB (5,5-dithiobis (2-nitrobenzoic acid)) were 0.039, 6.22 and 13.6, respectively. The assay conditions were as follows:

Superoxide dismutase (SOD; EC 1.15.1.1) activity was measured by the ferricytochrome c method using xanthine/xanthine oxidase as the source of superoxide radicals. The reaction mixture consisted of 50 mM potassium phosphate buffer (pH 7.8), 0.1 mM EDTA, 0.1 mM xanthine, 0.013 mM cytochrome c and 0.024 IU/mL xanthine oxidase. Activity is reported in units of SOD per milligram of protein. One unit of activity was defined as the amount of enzyme necessary to produce a 50% inhibition of the ferricytochrome c reduction rate [48].

Catalase (CAT; EC 1.11.1.6) activity was determined by measuring the decrease in hydrogen peroxide concentration at 240 nm according to Aebi [49]. The reaction mixture contained 50 mm potassium phosphate buffer (pH 7.0) and 10.6 mM freshly prepared H_2_O_2_.

Glucose-6-phosphate dehydrogenase (G6PDH; EC 1.1.1.49) activity was determined at pH 7.6 in a medium containing 50 mM HEPES buffer, 2 mM MgCl_2_, 0.8 mM NADP^+^ and glucose 6-phosphate used as substrate. The enzyme activity was determined by measuring the reduction of NADP^+^ at 340 nm as previously described by Lupiáñez et al. [2] and Peragón et al. [50]. The change in absorbance at 340 nm was recorded and, after confirmation of no exogenous activity, the reaction started by the addition of substrate.

Glutathione S-transferase (GST; EC 2.5.1.18) activity was measured according to the method described by Habig et al. [51], using 1-chloro-2,4-dinitrobenzene as a substrate.

Glutathione peroxidase (GPX, EC 1.11.1.9) activity was determined using the method described by Flohe and Günzler [52], based on the oxidation of NADPH, which is used to regenerate the reduced glutathione (GSH) from oxidized glutathione (GS-SG) obtained by the action of glutathione peroxidase.

Glutathione reductase (GR, EC 1.8.1.7) was determined by the modified method of Carlberg and Mannervik [53]. We measured the decrease in absorbance produced by the oxidation of NADPH used by GR in the passage of oxidized glutathione (GS-SG) to reduced glutathione (GSH).

All enzyme activities (except for SOD) are expressed as units or milliunits per milligram of soluble protein (specific activity). One unit of enzyme activity was defined as the amount of enzyme required to transform 1 µmol of substrate per min under the above assay conditions.

Soluble protein concentrations were determined using the method of Bradford, with bovine serum albumin used as a standard.

### 4.6. Statistical Analysis

Data are shown as mean ± standard error mean (SEM). The statistical significance among different experimental groups was determined by one-way analysis of variance (ANOVA) tests. When *F* values (*p* < 0.05) were significant, means were compared using Tukey’s HSD test. The SPSS version 15.0 for Windows software package was used for statistical analysis.

## 5. Conclusions

In conclusion, the results obtained in the present study demonstrate that MA exerts a selective anti-proliferative effect against the B16F10 cell line, whereas in A10 healthy cells, MA did not present a cytotoxic effect. This natural compound prevents the oxidative stress caused by H_2_O_2_ excess, decreasing the ROS production levels. Moreover, depending on the cell line, the antioxidant enzymatic responses were different in the presence of H_2_O_2_ and without MA. Thus, in healthy A10 cells, all enzymes up-regulated their activity, but in B16F10 cells, only SOD, GST and GPX increased it. In most cases, MA treatment restored the activities of enzymes to levels similar to those in the control group, highlighting that in A10 cells, the highest doses of MA (IC_50_ and 2·IC_50_) resulted in values below control. Overall, these findings demonstrate the great antioxidant capacity of maslinic acid.
